# Statin protects men but not women with HIV against loss of muscle mass, strength, and physical function: a pilot study

**DOI:** 10.1038/s41598-023-31643-3

**Published:** 2023-03-22

**Authors:** José David G. Cárdenas, Vitor H. F. Oliveira, Ana L. Borsari, Poliana C. Marinello, Chris T. Longenecker, Rafael Deminice

**Affiliations:** 1 Health Sciences Graduate Studies, State University of Londrina, Londrina, Paraná Brazil; 2 Department of Child, Family and Population Health Nursing, University of Washington, Seattle, Washington USA; 3 Department of Physical Education, State University of Londrina, Londrina, Paraná Brazil; 4 Biological Sciences Center, State University of Londrina, Londrina, Paraná Brazil; 5 Department of Cardiology and Global Health, University of Washington, Seattle, Washington USA; 6 Department of Physical Education, Faculty of Physical Education and Sport, State University of Londrina, Rodovia Celso Garcia Cid, Pr 445 km 380, Campus Universitário, Londrina, PR Brazil

**Keywords:** HIV infections, Musculoskeletal system

## Abstract

Statins are cholesterol-lowering drugs commonly used among people with HIV, associated with an increased risk of myopathies. Considering that cardiovascular disease, statin therapy, and sarcopenia are independently prevalent in people with HIV, clarity on the potential benefits or harms of statin therapy on muscle health is useful to provide insight into ways to maximize skeletal muscle health and minimize CVD risk in this population. We aimed to study the effects of statin therapy on strength, muscle mass, and physical function parameters in people with HIV. This was a pilot cross-sectional study. People with HIV on continuous statin therapy (*n* = 52) were paired 1:1 according to age (people with HIV 53.9 ± 8.2 and people with HIV on statins 53.9 ± 8.4 years), sex, body mass index (Body mass index, people with HIV 28.6 ± 5.3 and people with HIV on statins 28.8 ± 6.3 kg/m^2^), and race with people with HIV not using statin (*n* = 52). Participants were evaluated for muscle strength (i.e. handgrip strength), lean and fat body mass (using bioelectric impedance analysis), and physical function (i.e. Short Physical Performance Battery—SPPB). Isokinetic strength and appendicular lean mass (using dual-energy X-ray absorptiometry), more accurate strength and body composition measures, were determined in 38% of the participants. Overall, statin usage does not exacerbated loss of muscle strength (32.2 ± 11.5 vs. 30.3 ± 9.6 kg, *p* > 0.05) muscle mass (7.6 ± 1.8 vs. 7.7 ± 1.1 kg/m^2^, *p* > 0.05), and impaired physical performance (10.1 ± 1.8 vs. 9.7 ± 2.1 points, *p* > 0.05) of PLWH. When analyzed by sex, men living with HIV on statins usage presented higher appendicular muscle mass (28.4 ± 3.1 vs. 26.2 ± 4.9 kg, *p* < 0.05) handgrip strength (42.1 ± 8.8 vs. 37.1 ± 8.3 kg, *p* < 0.05) and physical function through SPPB score (10.9 ± 1.3 vs. 9.5 ± 2.1, *p* < 0.05) than men living with HIV not on statins treatment. The same protection was not observed in women. This data was demonstrated when muscle mass and strength were determined clinically (i.e. handgrip strength and electrical impedance) and when more precise laboratory measurements of muscle mass and strength were conducted (i.e. isokinetic strength and DXA scans). Statin does not exacerbate muscle wasting, strength loss, or muscle dysfunction among people with HIV. Indeed, statins may protect men, but not woman with HIV against HIV and antiretroviral therapy-induced loss of muscle mass and strength.

## Introduction

The implementation of antiretroviral therapy (ART) has turned HIV infection into a manageable and chronic condition. Indeed, people living with HIV (PLWH) currently experience an average life expectancy close to that of the general population^1^. With a longer life expectancy, PLWH are now experiencing a high incidence of aging-related comorbidities in addition to the ART side effects^2,3^. As a result, PLWH may present clinically with a range of metabolic disorders, cardiovascular disease (CVD), muscular atrophy, and dyslipidemia, among other comorbidities, earlier than people without HIV^1^.

CVD and dyslipidemia are highly prevalent among PLWH; CVD is the leading cause of death in this population, and studies have shown that the prevalence of dyslipidemia is approximately three times higher after ART initiation^4,5^. The increased CVD risk among PLWH is multifactorial and attributed to the interaction between traditional CVD risk factors (e.g. smoking, physical inactivity), persistent chronic inflammation, and immune activation^4,5^. Statins are first-line cholesterol-lowering agents in the general population for primary and secondary prevention of CVD^6^. Together with reducing cholesterol levels, studies have shown that statins have pleiotropic effects, including improving endothelial function, slowing the progression of atherosclerosis, stabilizing the atherosclerotic plaque, and reducing inflammatory biomarkers^7^. Indeed, improved lipid profiles and reduced all-cause mortality have been observed in PLWH on statin therapy^8^.

Although beneficial and usually well-tolerated, statin therapy has been linked to skeletal muscle side effects, including myalgia, cramps, muscle weakness, and changes in energy metabolism^6,9^. Indeed, studies have demonstrated statin-associated muscle symptoms (SAMS) may affect up to 29% of patients, becoming the main reason for statin therapy non-adherence or withdrawal^10,11^. In contrast, some recent data postulate that the frequency of SAMS may be overestimated and more associated with high doses of statins in the general population^12^. Furthermore, placebo-controlled trials demonstrate that most SAMS are not caused by statins but represent misattribution^13^. Moreover, SAMS and its consequences are poorly studies among PLWH. This is relevant since PLWH may experience muscle strength loss, muscle wasting, and impaired physical performance up to 10 years earlier than people without HIV^14–18^. Sarcopenia, a musculoskeletal disease characterized by progressive loss of skeletal muscle mass and function^19^, is highly associated with hospitalization and mortality rates in the general population. Sarcopenia has been recently demonstrated to be high prevalent among PLWH^20,21^. However, the relationship between statin therapy, skeletal muscle outcomes, and physical function is still unclear, especially among PLWH^22–24^.

Considering that CVD, statin therapy, and sarcopenia are independently prevalent in PLWH, clarity on the potential benefits or harms of statin therapy on sarcopenia-defining parameters is useful to provide insight into ways to maximize skeletal muscle health and minimize CVD risk in this population. Thus, this study aims to investigate the effect of statins on sarcopenia-defining parameters (i.e. muscle strength, muscle mass, and physical performance) in PLWH. Based in recent studies on statins SAMS, we hypothesized that statins do not worsen muscle strength, muscle mass, and physical performance in PLWH.

## Methods

### Participants

PLWH were recruited in southern Brazil between July 2018 and November 2019 at the Londrina State University Hospital and the Integrated Center for Infectious Diseases of Londrina. Participants were approached and invited to participate while waiting for their routine medical care. The following inclusion criteria were applied: (a) having a confirmed HIV-1 diagnosis noted in their medical records; (b) ≥ 18 years of age; (c) being prescribed ART; (d) not being on hormone replacement therapy or using anabolic drugs; (e) not having an advanced immunodeficiency status or active opportunistic infection; (f) having the cognitive and physical capacity to perform the tests.

All participants were informed about the procedures and activities to perform and signed a informed consent form before any procedure. The study protocol was approved by the Ethics Board Committee for Research Involving Human Subjects of Londrina State University (approval document #2.305.624) and was in accordance with the ethical guidelines of the Declaration of Helsinki. All study participants were volunteers and did not receive any kind of compensation.

### Study design

This is a descriptive pilot cross-sectional analysis of data from a study aiming to improve knowledge about sarcopenia among PLWH. All the tests and assessments performed were chosen according to the most recent consensus on sarcopenia operational definition and diagnosis^19^. The study was conducted in two phases. During the first phase, participants completed an interview on sociodemographic and medical information. After that, their body mass, height, body composition (i.e. bioelectric impedance analysis [BIA]), handgrip strength, and physical function (i.e. short physical performance battery [SPPB]) were determined in a single visit using tests suitable to the clinical setting. At the end of the visit, participants’ medical history was abstracted using chart review. During the second phase, all participants assessed in the first phase were invited to attend two additional visits to the Exercise Biochemistry Laboratory at Londrina State University. During these visits, participants’ muscle strength and muscle mass were determined using isokinetic dynamometry and dual-energy X-ray absorptiometry (DXA) scanning.

**Figure 1:**
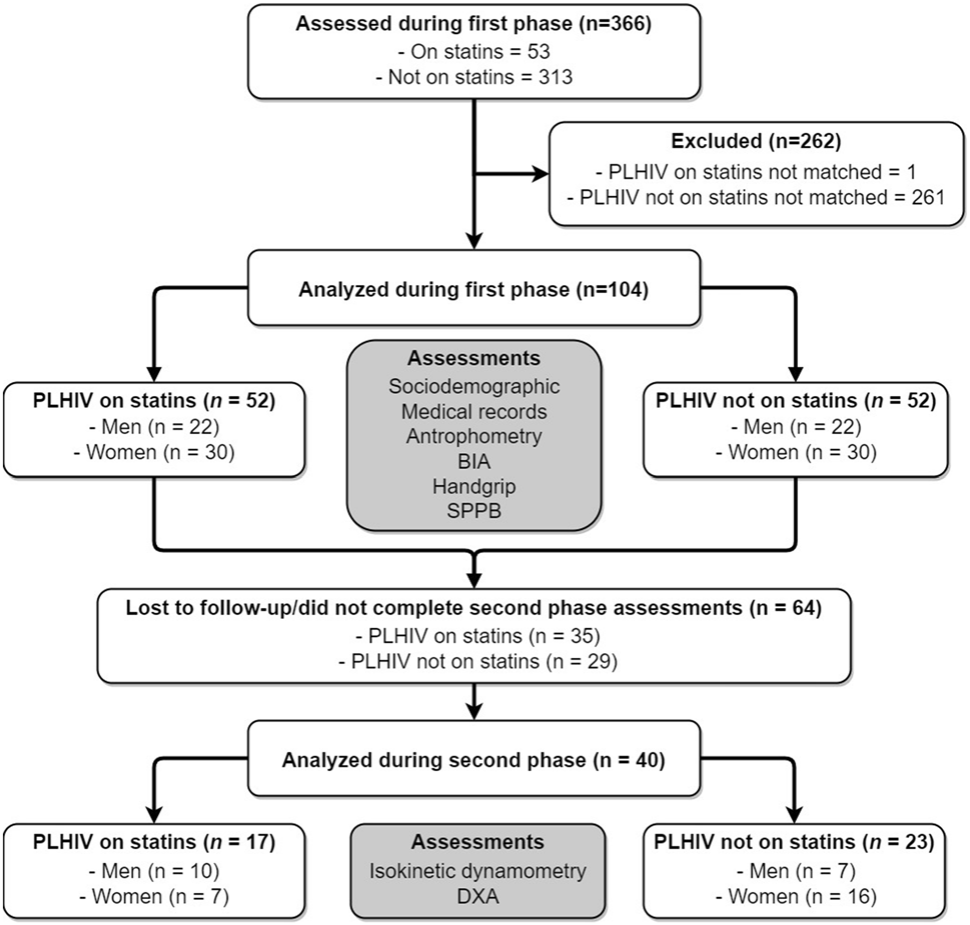
The flow chart of the study’s participants. *PLWH*   people living with HIV, *BIA*   bioelectric impedance analysis. In total, 366 participants were evaluated, 53 PLWH were identified using statins and were paired according to age, sex, body mass index and race with PLWH not using statins. Finally, 104 participants (52 per group) were included in the clinical determination of sarcopenia. Finally, 40 PLWH completed the DXA and isokinetic dynamometry evaluation. *DXA* dual-energy X-ray absorptiometry, *SPPB*   short physical performance battery.

During the first phase, 366 participants (189 men and 177 women, aged 47.9 ± 11.9 years) were evaluated. Among these, 53 PLWH were identified using statins and were paired according to age (± 1 year), sex, body mass index (BMI, ± 1 kg/m^2^), and race with PLWH not using statins. One of the participants on statins could not be matched according to our criteria and was therefore excluded. Finally, 104 participants (52 per group) were included in the analysis for the first phase. From the participants included in the first phase, 40 PLWH (17 on and 23 not on statins) agreed to participate in the second phase and completed the DXA and isokinetic dynamometry evaluation. The participants' flow is presented in Fig. 1.

### Study procedures

Demographic and clinical characteristics. Information on race and medical history was collected through interviews using a predetermined questionnaire. Additional medical information was obtained from the patients’ medical records, including the following: (a) laboratory results (i.e. total cholesterol, low-density lipoprotein [LDL], high-density lipoprotein [HDL], T-CD4 + and T-CD8 + lymphocytes); (b) dyslipidemia presence, time and drugs used for treatment; (c) current use and total duration of individual ART drugs and drug classes.

### Muscle strength

During the first phase, handgrip strength was measured with a hand-held dynamometer (SH 5001, Saehan Co., Siheung, South Korea). If the participant had recent hand surgery or current pain that could have influenced the results, the handgrip test was not performed. After the examiner explained how the dynamometer worked, demonstrated how the test should be performed, and adjusted the grip size of the dynamometer to the subject’s hand size, the test was performed with the participant seated in a chair and with the arm on the side of the body flexed at 90°. Two trials were attempted with each hand, and all results were recorded. The values presented were the mean of the best result of each hand. Verbal encouragement to motivate maximum effort during the test was consistent across participants. Low handgrip strength was considered when values were < 27 kg for men and < 16 kg for women^19^.

During the second phase, isokinetic strength tests were performed on a Biodex Multi-Joint System-PRO dynamometer (Biodex Medical Systems, Shirley, NY, USA) to obtain data on lower-limb muscle strength. After a five-minute warm-up in a cycle ergometer using a self-selected low intensity, the participants were positioned and stabilized in the dynamometer chair. The device was then calibrated, and the range of motion was set from 20° to 105°. Before the test, participants performed four submaximal repetitions to familiarize themselves with the test movement and techniques. The test was performed at speeds of 60°/s and 180°/s for knee extension and flexion in a concentric–concentric mode. The protocol included a set of five repetitions at a speed of 60°/s and a set of 30 repetitions at 180°/s, with a one-minute interval between sets. The test was performed only with the right leg, and the participant was continuously motivated to achieve maximum effort during the test. The isokinetic strength test was performed in two different sessions, the first one being a familiarization session and the second a testing session, occurring 1 week after the familiarization session and at the same time of day. The variable peak torque (absolute and normalized by body mass), average power, and total work were obtained.

### Anthropometry and body composition

Body mass and height were obtained according to the procedures described by Gordon et al.^25^. Body composition was estimated using a BIA device (Bia Analyzer, Rushford NanoElectroChemistry Co., Rushford, MN, USA) during the first phase, according to the procedures described by Sardinha et al.^26^. Before performing the test, the participants confirmed that they had refrained from physical activity during the previous 24 h. Participants were then asked to remove any metal accessories from the body, stay barefoot, and wear comfortable clothing. The procedure was conducted with subjects lying supine with their legs and arms apart, and four electrodes were placed on the right side of the body. The appendicular skeletal muscle mass (ASM) was estimated using the equation described by Kyle et al.^27^ and divided by the squared height, thus obtaining the appendicular skeletal muscle mass index (ASM/h^2^). Low muscle mass was considered when ASM/h^2^ was < 7.0 kg/m^2^ for men and < 5.5 kg/m^2^ for women^19^.

DXA scans were completed during the second phase on a Lunar Prodigy Advance (G.E. Medical Systems, Madison, WI, USA) in conjunction with enCORE software version 16.0. Scans were performed with participants lying flat, in light clothes, barefoot, and without having any metallic objects or other accessories next to their bodies. Equipment calibration followed the manufacturer’s recommendations, and an experienced laboratory technician performed both calibration and analysis.

### Physical function

The Short Physical Performance Battery—SPPB was used to assess physical function^28^. The SPPB is composed of three different tests: (a) static balance, consisting of positioning the feet in three different positions (i.e. standing with the feet together [side-by-side standing], standing with one foot partially in front [semi-tandem stand], and standing with one foot forward [tandem stand]), which should be held for up to 10 s; (b) chair stand test (five times sit-to-stand), consisting of getting up completely from the chair as quickly as possible five times in a row, without stopping between repetitions; and (c) gait speed test, consisting of walking a distance of four meters twice, with the fastest time being used to calculate the result (m/s). After performing the three tests, participants were classified according to the SPBB recommendations, scoring from 0 to 12. Low physical function was defined as an SPPB score ≤ 8 points^19^.

### Statistical analysis

Descriptive statistics are presented as mean ± standard deviation for continuous variables and prevalence (percentage) for categorical variables. Data normality was checked using the Shapiro–Wilk test and Levene’s test was used to analyze the homogeneity of variances. Independent *t* tests or Mann–Whitney tests were used to examine differences between PLWH on and not on statins for parametric and non-parametric data, respectively. In sex-stratified analyses, two-way ANOVA followed by the Bonferroni post hoc test or the Kruskal–Wallis test followed by Dunn’s post hoc test were used to examine differences between men and women with HIV on and not on statins when data were parametric and non-parametric, respectively. The Chi-square test was used to determine differences in contingency tables. All analyses were performed using IBM SPSS 26.0 (Armonk, NY, USA) and a p value ≤ 0.05 was considered significant.

### Ethics approval and consent to participate

All participants were informed about the procedures and activities to perform and signed a consent form before any procedure. The study was approved by the Ethics Board Committee for Research Involving Human Subjects of Londrina State University (approval document #2.305.624). All study participants were volunteers and did not receive any kind of compensation.

## Results

The characteristics of the study participants are presented in Table 1. A total of 52 PLWH on statins were included in the study and were paired with PLWH not on statins. Most participants were white (44%), followed by multiracial or indigenous (35%) and black (11%). There were no statistically significant differences (p > 0.05) between groups regarding HIV characteristics, although PLWH on statins tended to have been treated with ART for longer compared to those off statins. Interestingly, lipid fractions were also similar between groups except for a trend toward more extremely elevated LDL (> 190 mg/dL) among statin users. Atorvastatin was the most used statin agent (56%), followed by simvastatin (36%). Other statin agents (i.e. rosuvastatin and pravastatin) were used by 8% of the participants.

**Table 1:** Participants’ characteristics.

	PLWH not on statins (*n* = 52)	PLWH on statins (*n* = 52)	*p*
Age (years)	53.9 ± 8.2	53.9 ± 8.4	0.98
Body mass index (kg/m^2^)	28.6 ± 5.3	28.8 ± 6.3	0.94
Males/females (%)	22 (43)/30 (57)	22 (43)/30 (57)	1
Race			
White (%)	23 (44)	23 (44)	1
Multiracial or indigenous (%)	18 (35)	18 (35)	1
Black (%)	11 (21)	11 (21)	1
Alcohol and smoking			
Alcohol (%)	12 (23)	14 (27)	0.43
Smoking (%)	27 (52)	30 (57)	0.63
HIV characteristics			
Time living with HIV (months)	150.3 ± 94.9	161.8 ± 87.6	0.52
Time of ART use (months)	128.6 ± 80.6	136.7 ± .99.9	0.10
Detectable viral load (%)	2 (4)	4 (8)	0.33
CD4 + lymphocytes (cells/mm^3^)	662.6 ± 351.6	683.8 ± 308.1	0.74
CD8 + lymphocytes (cells /mm^3^)	1107.8 ± 446.7	1104.5 ± 490.4	0.97
CD4/CD8 ratio	0.6 ± 0.4	0.7 ± 0.4	0.52
ART regimen composition			
NRTI + PI (%)	25 (48)	26 (50)	0.89
NRTI + NNRTI (%)	15 (30)	20 (38.9)	0.57
NRTI + INSTI (%)	5 (10)	3 (6)	0.67
NRTI + PI + INSTI (%)	3 (6)	1 (2)	0.71
Other	4 (8)	2 (4)	0.43
Lipid fractions			
Total cholesterol (mg/dL)	202.3 ± 50.8	204.3 ± 51.2	0.90
Total cholesterol > 240 mg/dL (%)	7 (13)	8 (15)	0.88
HDL cholesterol (mg/dL)	44.5 ± 14.1	48.0 ± 18.5	0.76
HDL cholesterol < 40 mg/dL (%)	12 (23)	14 (27)	0.69
LDL cholesterol (mg/dL)	119.1 ± 44.2	121.2 ± 41.1	0.88
LDL cholesterol > 190 mg/dL (%)	2 (4)	4 (8)	0.12
Triglycerides (mg/dL)	197.5 ± 123.8	197.8 ± 102.2	0.93
Triglycerides > 200 mg/dL (%)	10 (19)	15 (29)	0.22
Statin use			
Atorvastatin (%)	–	29 (56)	–
Simvastatin (%)	–	19 (36)	–
Others (%)	–	4 (8)	–
Time on statin treatment (months)	–	49.9 ± 34.6	–

Values in the table are mean ± standard deviation when variables are continuous; and number and (%) when variables are categories. Continuous variables are expressed as mean ± standard deviation, and categorical variables are expressed as absolute (relative) values. Lipid fractions are “on treatment” lab values.

*PLWH* people living with HIV, *ART* antiretroviral therapy, *CD4* T-CD4 + lymphocytes, *CD8* T-CD8 + lymphocytes, *NRTI* nucleoside reverse transcriptase inhibitor, *PI* protease inhibitor, *NNRTI* nonnucleoside reverse transcriptase inhibitor, *INSTI* integrase strand transferase inhibitor, *HDL* high-density lipoprotein, *LDL* low-density lipoprotein.

Regarding the sarcopenia-defining parameters (i.e. muscle strength, muscle mass, and physical function) determined during the first phase, no statistically differences (p > 0.05) were observed for ASM/h^2^ (PLWH not on statins: 7.7 ± 1.1 vs. PLWH on statins: 7.6 ± 1.8 kg/m^2^), handgrip strength (PLWH not on statins: 30.3 ± 9.6 vs. PLWH on statins: 32.2 ± 11.5 kg), and SPPB total score or its components (total score = PLWH not on statins: 9.7 ± 2.1 vs. PLWH on statins 10.1 ± 1.8 points; balance = PLWH not on statins: 3.7 ± 0.8 vs. PLWH on statins: 3.8 ± 0.6 points; gait speed = PLWH not on statins 1.19 ± 0.16 vs. PLWH on statins: 1.24 ± 0.13 m/s; chair stand = PLWH not on statins 14.4 ± 5.7 vs. PLWH on statins 13.7 ± 5.6 s). On the other hand, some statistically differences were observed when the sample was stratified by sex (Table 2). Results demonstrated that men living with HIV on statins usage presented statistically significant higher ASM, handgrip strength, and SPPB score than men living with HIV not on statins. These results were not observed in women. Additionally, not statistically differences in age, BMI, time living with HIV, time of ART use, and fat mass were observed between men or women living with HIV on statins compared to their counterparts not on statins.

**Table 2:** Sarcopenia defining parameters in men and women living with HIV on and not on statins.

	Men			Women		
	PLWH not on statins (*n* = 22)	PLWH on statins (*n* = 22)	*p*	PLWH not on statins (*n* = 30)	PLWH on statins (*n* = 30)	*p*
Age (years)	53.5 ± 7.9	53.4 ± 8.0	0.98	54.2 ± 8.5	54.3 ± 8.8	0.95
Body mass index (kg/m^2^)	26.9 ± 2.3	29.1 ± 5.2	0.07	29.9 ± 6.4	28.4 ± 7.0	0.78
Time living with HIV (months)	144.2 ± 99.0	165.1 ± 88.4	0.47	154.6 ± 90.1	159.3 ± 88.5	0.88
Time of ART use (months)	122.5 ± 79.1	154.1 ± 83.2	0.20	132.8 ± 82.7	150.7 ± 90.3	0.78
Time under statins (months)	–	53.4 ± 43.2		–	45.3 ± 38.4	0.47^#^
Body composition						
Fat mass (kg)	20.5 ± 5.7	24.5 ± 12.0	0.19	29.2 ± 12.2	25.7 ± 11.2	0.29
Fat mass (%)	26.2 ± 4.9	27.5 ± 7.8	0.51	39.7 ± 7.4	36.3 ± 6.8	0.09
ASM (kg)	26.4 ± 1.9	28.4 ± 3.1	**0.04**	20.4 ± 4.1	18.9 ± 4.7	0.21
ASM/h^2^ (kg/m^2^)	8.5 ± 0.5	8.9 ± 1.1	0.09	7.1 ± 1.0	6.7 ± 1.7	0.37
Low ASM/h^2^ (%)	0 (0)	1 (4)	0.92	2 (7)	4 (13)	0.46
Muscle strength						
Handgrip Strength (kg)	37.1 ± 8.3	42.1 ± 8.8	**0.05**	24.6 ± 5.4	24.7 ± 6.5	0.93
Low Handgrip Strength (%)	2 (9)	1 (3)	0.48	2 (6)	2 (6)	1
Physical function						
Balance (score)	3.8 ± 0.7	3.8 ± 0.5	0.98	3.6 ± 0.8	3.7 ± 0.6	0.87
Gait speed (m/s)	1.27 ± 0.19	1.07 ± 0.16	** < 0.01**	1.22 ± 0.20	1.28 ± 0.17	0.21
Chair stand (s)	14.4 ± 4.8	11.9 ± 3.5	**0.05**	14.4 ± 6.4	14.5 ± 4.8	0.98
SPPB (score)	9.5 ± 2.1	10.9 ± 1.3	**0.01**	9.8 ± 2.1	9.6 ± 2.0	0.91
Low SPPB score (%)	6 (27)	2 (6)	**0.03**	8 (26)	6 (20)	0.65

Values in the table are mean ± standard deviation when variables are continuous; and number and (%) when variables are categories. Continuous variables are expressed as mean ± standard deviation, and categorical variables are expressed as absolute (relative) values. Low values for ASM/h^2^, handgrip strength, and SPPB were determined according to Cruz-Jentoft et al*.*^19^.

*PLWH* people living with HIV, *ART* antiretroviral therapy, *ASM* appendicular skeletal muscle mass, *ASM/h*^2^ ASM index, *SPPB* short physical performance battery.

^#^Comparison between men and women on statins.

Significant values are in bold.

Using the body composition measurements from DXA scans and isokinetic muscle strength variables obtained during the second phase (Table 3), PLWH on statins had statistically significant higher lean soft mass and fat-free mass, and lower relative fat mass compared to PLWH not on statins. Additionally, PLWH on statins had higher isokinetic strength compared to PLWH not on statins when the test was performed at 180°/s, demonstrated by higher absolute values and normalized by body mass of peak torque and total work. No differences in muscle strength were observed when the test was performed at 60°/s.

**Table 3:** Body composition by dual-energy X-ray absorptiometry scanning and muscle strength by isokinetic dynamometry measured in people living with HIV on and not on statins.

	PLWH not on statins (*n* = 23)	PLWH on statins (*n* = 17)	*p*
Body composition			
Total body mass (kg)	74.1 ± 17.5	77.7 ± 21.5	0.52
Lean soft mass (kg)	41.0 ± 9.3	49.7 ± 13.7	**0.02**
Fat free mass (kg)	43.7 ± 10.66	52.4 ± 14.2	**0.03**
Total fat mass (kg)	27.7 ± 7494.6	27.2 ± 11.1	0.78
Total fat mass (%)	40.2 ± 7.7	35.3 ± 7.7	**0.05**
ASM/h^2^ (kg/m^2^)	7.3 ± 1.1	8.0 ± 1.7	0.13
Muscle strength			
Peak torque @ 60°/s (N/m)	105.0 ± 42.3	125.8 ± 43.3	0.15
Peak torque/BW @ 60°/s (%)	142.5 ± 49.5	160.4 ± 51.6	0.28
Total work @ 60°/s (J)	448.6 ± 186.8	537.7 ± 167.8	0.16
Total work/BW @ 60°/s (%)	135.3 ± 48.1	155.1 ± 46.1	0.20
Peak torque @ 180°/s (N/m)	55.2 ± 22.9	75.9 ± 29.9	**0.01**
Peak torque/BW @ 180°/s (%)	75.3 ± 27.3	94.9 ± 27.4	**0.03**
Total work @ 180°/s (J)	1384.9 ± 692.2	1812.6 ± 669.5	**0.05**
Total work/BW @ 180°/s (%)	84.4 ± 32.3	104.6 ± 31.8	**0.04**

Values are expressed as mean ± standard deviation.

*PLWH* people living with HIV, *ASM/h*^2^ appendicular skeletal muscle mass index, *BW* total body mass.

Significant values are in bold.

**Figure 2:**
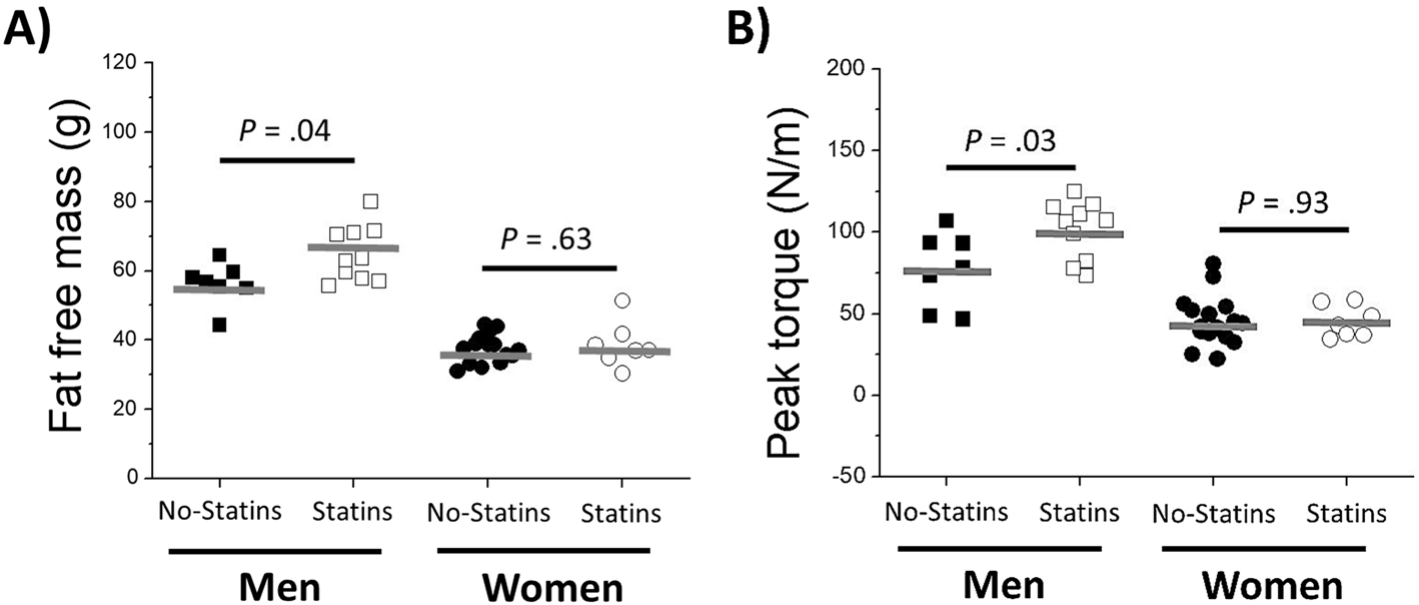
Men but no women living with HIV under statin treatment presented higher fat-free mass and strength comped with PLWH not on statins. (**A**) fat-free mass and (**B**) peak torque at 180°/s among men and women with HIV on and not on statins. Statins protected men, but not women from low fat fee mass and strength. Values are expressed as mean ± standard deviation. *p* < 0.05, compared with no-statins usage group by test *t* student.

When DXA and isokinetic muscle strength results were separated by sex (Fig. 2), men living with HIV under statin treatment presented higher fat-free mass and peak torque than men with HIV not on statins, a difference which was not observed for women.

## Discussion

The main findings of our pilot study were that overall, statins do not worsen muscle strength, muscle mass, and physical performance in PLWH. Moreover, considering all limitations of a pilot study, our data demonstrated statin therapy seems to be associated with more muscle mass and strength and better physical function in men and not women with HIV. Indeed, men with HIV using statins had higher muscle mass, strength, and better physical performance than men with HIV not on statins. These results were observed when muscle mass and strength were determined using tests suitable to the clinical setting (i.e. handgrip strength and BIA) and when more precise laboratory measurements were made (i.e. isokinetic strength and DXA scans). Similar results were not observed in women living with HIV or when men and women were analyzed together. These findings are relevant given that, to date, no pharmacological treatment has yet been approved for sarcopenia. Statis are relative cheap and well tolerated by PWH, a promising drug to treat sarcopenia.

Statins are of clinical importance for PLWH in the ART era, considering that dyslipidemia and CVD are highly prevalent in this population^4,5^. Although early studies reported different SAMS and raised concerns regarding the skeletal muscle health of statin users^6,9^, recent studies have demonstrated that statin therapy does not impair exercise performance, muscle strength, and physical activity in the general population, even in patients experiencing myalgia^29–31^. Among PLWH, the relationship between statin therapy and skeletal muscle outcomes is under-investigated and still unclear. A recent study of Erlandson et al.^23^ analyzed the effects of 96 weeks of rosuvastatin on bone, muscle, and fat in PLWH (147 participants [115 males], 72 receiving rosuvastatin and 75 receiving placebo), and they reported a trend for increased lean body mass in the rosuvastatin arm (0.8% increase versus 0.5% decrease in the placebo arm). Abdo et al.^22^ determined the association between statin use and age-associated changes in physical function among men with (n = 1048) and without HIV (n = 973) and reported that consistent statin users do not seem to have a major impact on physical function (i.e. gait speed and handgrip strength). More recently, Zanetti et al.^24^ evaluated the effects of the combination of exercise training and statins in PLWH and observed no difference in reduction of fat mass and increase in lean body mass, muscle strength, and cardiorespiratory fitness between the exercise/statins and exercise/placebo groups. Although the studies are small, these limited data seem to suggest that statin therapy does not negatively affect skeletal muscle health or physical function of PLWH and may even improve it.

PLWH have impaired physical function, decreased muscle strength, and muscle wasting when compared with people without HIV^14–18^ and thus increased attention has recently been given to sarcopenia among PLWH. Sarcopenia is a musculoskeletal disease characterized by progressive loss of skeletal muscle mass and function, and it is recognized as an important aging-related condition associated with chronic diseases and higher mortality rates in the general population^19^. Low muscle strength, mass, and physical performance have been considered the defining parameters of sarcopenia^19^. A recent meta-analysis carried out by our study group showed that sarcopenia is highly prevalent (24.1%) in PLWH, who were 6.1 times more likely to develop sarcopenia than people without HIV^21^. In addition to aging, sarcopenia among PLWH is caused by different factors linked to HIV management, such as mitochondrial dysfunction, persistent inflammation, and formation of reactive oxygen species^20^, in a phenomenon called “premature” or “accelerated” aging^32^. Some behavioral and lifestyle factors also play a role, where PLWH present a high prevalence of physical inactivity, smoking, and food insecurity^20,33,34^. Although under investigated, we believe that sarcopenia is an emerging health-related condition affecting PLWH, which will face the burden of aging-related sarcopenia and HIV management in the years to come. Considering that high levels of inflammatory markers are associated with sarcopenia development^35^, the pleiotropic anti-inflammatory effects of statin therapy may confer skeletal muscle benefits in populations with an increased risk of developing sarcopenia^36^. Statins may reduce reactive oxygen species in the endothelial tissue, increase the release of nitric oxide that improves endothelial function, inhibit the release of inflammatory cytokines (e.g. IL-1β) in the endothelium, and reduce C-reactive protein levels^7,37^. All these mechanisms may promote skeletal muscle health in PLWH.

Interestingly, differences were not observed among women of the same age, BMI, race, time living with HIV, and time of ART use on and not on statins. Sex-based differences are observed in HIV pathogenesis^38,39^, HIV related comorbidities such as cardiovascular disease^40,41^, and muscle strength among PLWH^42^. Scully^38^ demonstrated that female sex is associated with better control of HIV through heightened levels of immune activation, but this may be maladaptive over time as it may predispose to cardiovascular disease and other non-AIDS comorbidities. Indeed, women may have a higher burden of HIV related coronary artery disease and are more likely to have HIV-related heart failure^40,43^. Additionally, the cholesterol-lowering effects of atorvastatin are greater in women^44^, and it is suggested that women may be at greater risk for statin-related myotoxicity compared with men^45^. However, the sex differences and mechanisms on statin-associated effects on skeletal muscle are still unclear, requiring further research.

Despite the potential benefits of statin therapy, attention should be given to SAMS, the most prevalent adverse effects associated with statin therapy and one of the main reasons for statin non-adherence or discontinuation^6,9^. Statin side effects depend on different factors, such as drug class, dose, age, gender, presence of comorbidities, and co-treatment with certain drugs^46^. For PLWH, this is further complicated by interactions with ART. The U.S. Food and Drug Administration has released a safety communication alerting on the interactions (i.e. drug–drug interaction and recommended dose) between certain HIV drugs and statins to decrease the risk of muscle injury among PLWH^47^. Careful attention should be given to these interactions to prevent SAMS among PLWH.

Among the limitations of this pilot study, we include the lack of an HIV-uninfected control group, the cross-sectional design, the small sample size, no information on statin adherence, and no measures of inflammation. It is important to consider moreover, that sarcopenia is an emerging problem among PLWH^20^. A recent study demonstrated PLWH presented 6.1 greater odds (95% CI  1.1–33.5) of sarcopenia compared with people without HIV, matched by age, sex, BMI, and ethnicity; the estimated prevalence of sarcopenia among PLWH was 24.1% versus 10% among people without HIV^21^. Thus, although with a small sample size, this study suggests that statin therapy may confer some protection to men living with HIV against HIV- and ART-associated skeletal muscle impairments and sarcopenia development. This can help the design of larger studies and trials on the topic.

## Conclusion

In conclusion, statin therapy among PLWH does not exacerbate loss of muscle strength, muscle mass, and impaired physical performance, all of which are key parameters of sarcopenia development. Notably, it seems that statins may improve these parameters among men living with HIV. Considering our is a pilot study, further research is needed in a larger cohort to determine whether statins might improve skeletal muscle parameters and whether there are sex differences.

## Data Availability

The datasets generated and/or analyzed during the current study are not publicly available but may be made available upon reasonable request from the corresponding author subject to applicable policies of the organizations that the authors are affiliated.
